# Mucocoele of the appendix

**DOI:** 10.4314/gmj.v58i1.15

**Published:** 2024-03

**Authors:** Ernest Oyeh, Josephine Nsaful, Antoinette Bediako-Bowan, Hafisatu Gbadamosi, Yaw Boateng Mensah, Nii A Adu-Aryee, Veneranda Nyarko

**Affiliations:** 1 Akai House Clinic, Accra, Ghana; 2 Department of Surgery, University of Ghana Medical School, College of Health Sciences, University of Ghana; 3 Department of Surgery, Korle Bu Teaching Hospital; 4 Department of Radiology, Korle Bu Teaching Hospital; 5 Department of Radiology, University of Ghana Medical School, College of Health Sciences, University of Ghana; 6 Department of Pathology, Korle Bu Teaching Hospital

**Keywords:** Mucocoele, Appendix, Hemicolectomy, Mucinous, Pseudomyxoma peritonei

## Abstract

**Introduction:**

Mucocoele of the appendix occurs in 0.2-0.7% of people in the world without any well-defined clinical symptoms. It occurs when there is an accumulation of mucous in the lumen of the appendix.

**Case Presentation:**

We present three cases: a 48-year-old male admitted to the emergency room with a one-day history of right iliac fossa pain. Abdominal examination was suggestive of acute appendicitis. The initial abdominal computerised tomography scan was reported as being unremarkable. At surgery, a firm tumour of the appendix was found, and a limited right hemicolectomy was done. Histopathology confirmed a mucocoele of the appendix with borderline mucinous histology.

The second case is a 63-year-old man who presented with a one-year history of abdominal distension and weight loss. Previous abdominal ultrasound was suggestive of liver cirrhosis with significant ascitic fluid. Abdominal magnetic resonance imaging found an appendix mucocoele with infiltration of the omentum and scalloping of the liver surface suggestive of pseudomyxoma peritonei. A percutaneous biopsy of the omental mass confirmed metastatic mucinous adenocarcinoma of the appendix.

The third case is a 68-year-old man who, during an annual medical check-up, had an incidental finding of a cystic right iliac fossa mass on ultrasound, confirmed on abdominopelvic computerised tomography scan to be an appendix mucocele. He had laparoscopic appendicectomy. The histopathological diagnosis confirmed a mucinous cystadenoma of the appendix.

**Conclusion:**

Preoperative diagnosis of appendiceal mucocoele is difficult and commonly discovered intraoperatively. The prognosis is good for the histologically benign type, but it is poor when malignant or peritoneal lesions are present.

**Funding:**

None declared

## Introduction

Mucocoele of the appendix is rare, occurring in 0.2-0.7% of people worldwide with non-specific symptoms. The appendiceal lumen is obstructed and distended with mucous. It commonly occurs over 50 years of age with conflicting sex preponderance.^1^

Four histological types have been described based on the nature of the differentiation of the appendiceal epithelial lining: the retention cyst, mucosal hyperplasia, mucinous cystadenoma and mucinous cystadenocarcinoma.^2^ WHO has also classified it into neoplastic (mucinous adenoma, low-grade appendiceal mucinous neoplasm (LAMN) and appendiceal adenocarcinoma) and non-neoplastic (mucosal hyperplasia, simple cyst).^3^ Grossly, retention cysts have transverse appendiceal diameters less than 2 cm, whilst mucosal hyperplasia, mucinous cystadenoma and mucinous cystadenocarcinoma have transverse diameters greater than 2cm.^4^ The incidence of this condition has not been reported in Ghana. We present three cases of appendiceal mucocoele with different presentations.

## Case Presentaion

### Case 1

A 48-year-old male reported to the emergency with a one-day history of lower abdominal pain that radiated to his right iliac fossa and was associated with anorexia, low grade fever, chills and passage of two loose stools. On abdominal examination, there was tenderness, rebound tenderness and guarding at the right iliac fossa. He had a non-contrast computerised tomography (CT) scan of the abdomen and pelvis which was initially reported as being normal prior to surgery. Laboratory investigations showed leucocytosis with neutrophilia.

**Figure 1:**
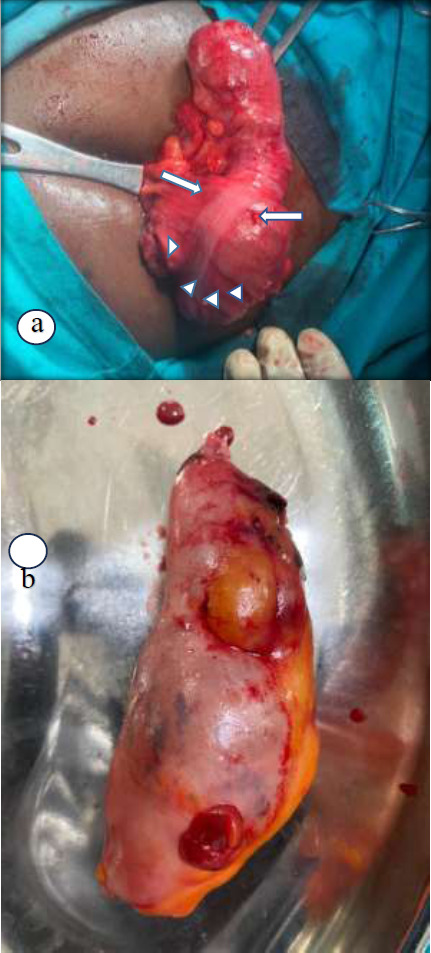
Intraoperative pictures of appendix. **a.** Huge tumour of the appendix for case 1 with long arrow at base of the appendix and arrowhead showing tumour in the lumen of the caecum. **b.** Tumour involving the body and tip of the appendix with a grossly normal base for case 3

A diagnosis of acute appendicitis was made and an appendicectomy via a lanz incision was done. A 12 x 5cm firm tumour of appendix extending beyond the base into the lumen of the caecum was found (figure1a). There was no perforation of the appendix, no enlarged mesenteric lymph nodes, and no peritoneal lesions. The lanz incision was closed up and surgery converted to a laparotomy. A limited right hemicolectomy was performed. Histopathological diagnosis confirmed a mucocoele of the appendix with borderline mucinous histology.

On post-operative day 2, the patient's abdomen was noticed to be distended with absent bowel sounds. A diagnosis of ileus was made. A nasogastric tube was passed and after persistent ileus for a further 2 days, a re-laparotomy was done which found a loop of small bowel adherent to a pin-point anastomotic dehiscence. An ileostomy was done. Three months later, he had a laparotomy to reverse the ileostomy. Adhesiolysis was done with end-to-end ileocolic anastomosis. There were no mucinous substances on the peritoneum. He is doing well and is scheduled for his first surveillance colonoscopy one year after the initial surgery.

### Case 2

A 63-year-old man presented to the emergency room a one-year history of progressive abdominal distension, weight loss, and anorexia. On physical examination, he was found to be significantly cachectic with a distended abdomen. Significant laboratory workup revealed a hemoglobin of 9.3g/dL, serum albumin 27g/L (35-52) and a positive Hepatitis B surface antigen. He had had several abdominopelvic ultrasound scans prior to presentation which reported the presence of gross ascites and cirrhosis of the liver. A repeat ultrasound scan on admission corroborated the presence of gross ascites without sonographic evidence of liver cirrhosis however, a right flank mass was noticed.

**Figure 3:**
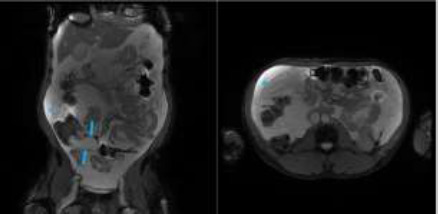
Coronal axial T2 weighted with fat suppression Magnetic Resonance Images of the abdomen and pelvis showing fluid-filled tubular dilatation of the appendix (long arrows), with associated infiltration of the omentum (short arrows) and gross ascites

A follow-up magnetic resonance image (MRI) of the abdomen and pelvis revealed the presence of tubular cystic dilatation of the appendix which measured 6.3 cm in length and 3.1cm in width, with no enhancing mass seen within it, Figure 3. Additionally, there was a heterogeneously enhancing omental mass in the right flank overlying the ascending colon as well as significant ascitic fluid. The ascitic fluid scalloped the margins of the liver and spleen, raising concerns for pseudomyxoma peritonei from a malignant appendix mucocele, with omental metastasis. There were well defined cystic lesions in the liver and spleen which appeared benign. Ultrasound-guided biopsy of the omental mass yielded histological findings consistent with metastatic mucinous adenocarcinoma of the appendix, with the ascitic fluid aspirate being positive for malignant cells. Unfortunately, the patient had a low-performance status precluding the start of chemotherapy. The patient is alive and receiving palliative care.

### Case 3

**Figure 4:**
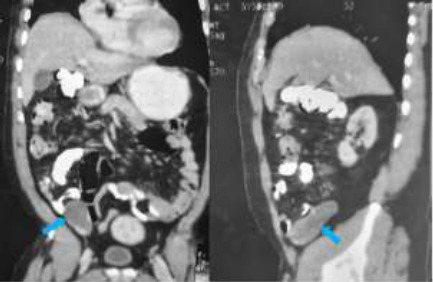
Contrast coronal, and sagittal reformatted CT images showing hypodense tubular dilatation of the appendix (blue arrows). No discernable enhancing focus within the dilated appendix

A 68-year-old man, as part of his annual medical checkup, had an ultrasound of his abdomen and pelvis, which showed a dilated cystic tubular lesion in the right iliac fossa. He had no gastrointestinal symptoms and examination findings were normal. He was a known hypertensive and asthmatic. He had been managed for sarcoidosis 10 years prior to presentation. Follow-up abdominopelvic CT imaging demonstrated cystic dilatation of the blind ending appendix which measured 9.0cm long and 3.3 cm in width consistent with an appendix mucocele as shown in Figure 4. Calcific foci were also seen within the appendix; however, no demonstrable luminal enhancement was present to suggest the presence of a polyp or neoplasia.

**Figure 5:**
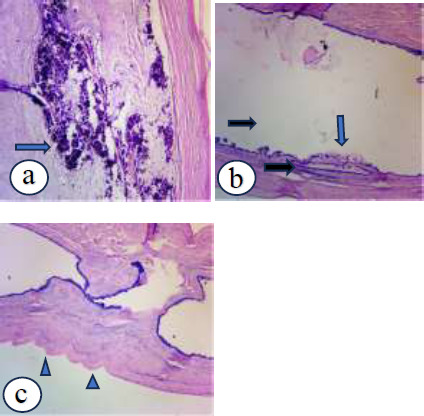
Histopathology of mucocele for Case 3. Arrow in a showing area of calcification within lumen. Black arrows in b showing multiple cysts with marked cystic dilatation filled with mucin and blue arrow showing epithelial lining composed of bland mucinous columnar cells. Arrow heads in c showing the thickened wall

He had laparoscopic appendicectomy. The findings were a tumour involving the body and tip of the appendix with a grossly normal base (figure 1b). The rest of the abdomen looked normal. Histopathology findings were a grossly distended 10.5x4.2x4cm appendix containing mucinous material. Sections of the appendix showed marked cystic dilation with thickened wall, lined by bland mucinous epithelium and filled with mucin (figure 5). The wall was partly hyalinized and calcified. The histopathological diagnosis was a mucinous cystadenoma. A surveillance colonoscopy done a year after was normal, and CEA levels have remained low.

## Discussion

Mucocele of the appendix was first described in the late 19^th^ century.^5^ It occurs as a cystic dilatation of the appendix due to mucus accumulation in the lumen by either inflammation, intraluminal neoplastic proliferation, or caecal lesion close to the appendiceal ostium.^6^

Clinical presentation is not clear-cut. For some, it presents as a right lower quadrant or vague lower abdominal pain, anorexia, vomiting, weight loss, gastrointestinal bleeding. In others, it presents as an abdominal mass or appendiceal intussusception that confounds the clinical picture with differentials of appendicitis or a tubo-ovarian mass in the female patient.^5^ In a large retrospective study by Yakan et al on 2120 patients with mucocoeles, the commonest clinical complaint was right lower quadrant pain and though this was a major symptom for our first patient, it still remains a nonspecific feature of the disease.^7^ It may also present as an incidental intraoperative finding.^5^ Notable complications include intestinal obstruction or bleeding, melaena, torsion, intussusception, ureteric obstruction, haematuria, pyonephrosis and as in the second case, pseudomyxoma peritonei which has a poor prognosis.^8^,^9^

Several other medical conditions may mimic appendiceal mucocoele posing a diagnostic challenge. In general, conditions such as appendicitis, peri-appendiceal abscess, diverticulitis, inflammatory bowel disease, mesenteric ischaemia, lymphocoele, peritoneal inclusion cyst, pyelonephritis, urolithiasis, cystitis, inguinal hernia and appendiceal neoplasms (carcinoid, adenocarcinoma, lipoma, fibroma, leiomyoma) are to be excluded. Additionally, uterine fibroid, adenomyosis, pelvic inflammatory disease, tubo-ovarian abscess, hydrosalpinx, ruptured ovarian cyst and endometriosis should be excluded in females whilst benign prostatic hypertrophy should be excluded in males preoperatively.^1^,^10^,^11^

Imaging is critical to avoid misdiagnosis and delayed diagnosis which can avert rupture leading to pseudomyxoma peritonei.^12^ Appendiceal mucocele can be diagnosed with ultrasound, CT scan and colonoscopy. Ultrasound, which incidentally identified the cystic appendix in this third case, is the first line investigation in acute abdominal presentations and can assist in distinguishing appendiceal mucocele from acute appendicitis with a sensitivity of 83% and specificity of 92% 10 An outer diameter of 6mm and 15mm or more indicates acute appendicitis and appendiceal mucocele respectively. The presence of surrounding inflammatory changes like fat stranding or reactive lymph nodes is indicative of acute appendicitis. Appendiceal mucocele appears oval or pear-shaped and has an onion-skin appearance due to the echoes of concentric layers of mucin. 50% of cases cast acoustic shadows due to dystrophic mural calcifications.^1^,^10^

CT scan is used to study the extent of the disease, identify underlying neoplasm and also confirm specific diagnostic features of appendiceal mucocele which appears as a dilated blind-end tubular structure continuous with the caecum filled with homogenous low-echogenic contents, curvilinear mural calcifications and a diameter more than 1.3 cm. 1,5 In case 1, the abdominal CT scan showed an appendiceal mucocoele measuring 4.5cm.

Colonoscopy reveals elevated appendicular orifice oozing yellowish mucous discharge and may detect associated colonic neoplasm. Fine needle aspiration cytology is not advised due to the risk of perforation and subsequent seeding in the peritoneal cavity.^5^

Surgical intervention, either open surgery or laparoscopic surgery, is required for the treatment of appendiceal mucocele. And this choice depends on whether the appendiceal mucocoele is perforated or not, involvement of the base of appendix and the presence of mesoappendix and ileocolic lymph nodes. A normal caecum with neither involvement of base of appendix nor lymph node involvement or perforation of the appendix, could have a simple appendectomy, whilst involvement of the base of appendix, lymph nodes and a perforation would benefit from right hemicolectomy.^13^ In case 1, there was extension beyond the base of the appendix into the lumen of the caecum and hence a right hemicolectomy was done.

Pseudomyxoma peritonei is rare sequelae which occurs when there is spontaneous rupture of the mucocoele resulting in intraperitoneal spread and implantation of mucinous cells causing a gelatinous ascites as in case 2. This may also arise from iatrogenic rupture during surgery. Occasionally it may be found extra peritoneally in the pleura.^14^ Treatment is with cytoreductive surgery and hyperthermic intraperitoneal chemotherapy (HIPEC) with mitomycin C. Recurrence is common, and the prognosis is poor.^15^

Follow-up with surveillance colonoscopy is recommended as there is an associated risk of colonic tumours. Carcinoembryonic antigen (CEA), CA19-9, and CA-125 are the tumour markers for follow-up, the elevation of which may herald disease recurrence.^16^,^17^ Case 1 is awaiting his first surveillance colonoscopy, and case 3 had normal colonic findings at his first surveillance colonoscopy, a year later.

## Conclusion

In conclusion, although rare, appendiceal mucocoele presents with diagnostic difficulty due to its non-specific symptoms and requires accurate diagnosis preoperatively to enable timely and careful surgery to avoid the dreaded complication of pseudomyxoma peritonei. Ultrasound and CT scan are important in establishing a diagnosis, and a definite diagnosis is achieved with histopathology of the surgical specimen. In all, the long-term prognosis of benign appendiceal mucocele is good but poor when malignant or ruptured.

## Figures and Tables

**Figure 2:**
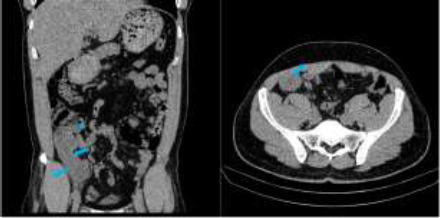
Non contrast coronal reformatted and axial CT scans of the abdomen and pelvis, demonstrating a large blind-ending tubular hypodense mass (long arrows), continuous with the caecum (short arrow) and consistent with a grossly dilated appendix
